# Cell-mediated immune response and protective efficacy of porcine reproductive and respiratory syndrome virus modified-live vaccines against co-challenge with PRRSV-1 and PRRSV-2

**DOI:** 10.1038/s41598-020-58626-y

**Published:** 2020-02-03

**Authors:** Adthakorn Madapong, Kepalee Saeng-chuto, Alongkot Boonsoongnern, Angkana Tantituvanont, Dachrit Nilubol

**Affiliations:** 1 Department of Veterinary Microbiology, Faculty of Veterinary Science, Chulalongkorn University, Bangkok, Thailand; 2 Department of Farm Resources and Production Medicine, Faculty of Veterinary Medicine Kamphaeng Saen Campus, Kasetsart University, Nakon Pathom, Thailand; 3 Department of Pharmaceutics and Industrial Pharmacy, Faculty of Pharmaceutical Sciences, Chulalongkorn University, Bangkok, Thailand

**Keywords:** Vaccines, Live attenuated vaccines

## Abstract

Cell-mediated immunity (CMI), IL-10, and the protective efficacy of modified-live porcine reproductive and respiratory syndrome virus (PRRSV) vaccines (MLV) against co-challenge with PRRSV-1 and PRRSV-2 (HP-PRRSV) were investigated. Seventy, PRRSV-free, 3-week old, pigs were allocated into 7 groups. Six groups were intramuscularly vaccinated with MLV, including Porcilis (PRRSV-1 MLV, MSD Animal Health, The Netherlands), Amervac (PRRSV-1 MLV, Laboratorios Hipra, Spain), Fostera (PRRSV-2 MLV, Zoetis, USA), Ingelvac PRRS MLV and Ingelvac PRRS ATP (PRRSV-2, Boehringer Ingelheim, USA), and Prime Pac PRRS (PRRSV-2 MLV, MSD Animal Health, The Netherlands). Unvaccinated pigs were left as control. Lymphocyte proliferative response, IL-10 and IFN-γ production were determined. At 35 days post-vaccination (DPV), all pigs were inoculated intranasally with 2 ml of each PRRSV-1 (10^5.4^ TCID_50_/ml) and PRRSV-2 (10^5.2^ TCID_50_/ml, HP-PRRSV). Following challenge, sera were quantitatively assayed for PRRSV RNA. Pigs were necropsied at 7 days post-challenge. Viremia, macro- and microscopic lung lesion together with PRRSV antigen presence were evaluated in lung tissues. The results demonstrated that, regardless of vaccine genotype, CMI induced by all MLVs was relatively slow. Increased production of IL-10 in all vaccinated groups was observed at 7 and 14 DPV. Pigs in Amervac, Ingelvac MLV and Ingelvac ATP groups had significantly higher levels of IL-10 compared to Porcilis, Fostera and Prime Pac groups at 7 and 14 DPV. Following challenge, regardless to vaccine genotype, vaccinated pigs had significantly lower lung lesion scores and PRRSV antigens than those in the control group. Both PRRSV-1 and PRRSV-2 RNA were significantly reduced. Prime Pac pigs had lowest PRRSV-1 and PRRSV-2 RNA in serum, and micro- and macroscopic lung lesion scores (*p* < 0.05) compared to other vaccinated groups. In conclusion, PRRSV MLVs, regardless of vaccine genotype, can reduce viremia and lung lesions following co-challenge with PRRSV-1 and PRRSV-2 (HP-PRRSV). The main difference between PRRSV MLV is the production of IL-10 following vaccination.

## Introduction

Porcine reproductive and respiratory syndrome (PRRS) is a devastating disease in pigs characterized by reproductive and respiratory failures. PRRS virus (PRRSV), an enveloped, positive-sense single-stranded RNA virus belonging to the *Arteriviridae* family, order *Nidovirales*, is the causative agent^1^. Two antigenically distinct genotypes of PRRSV, PRRSV-1 and PRRSV-2, have been recognized. The genomes of both genotypes are 15 kb in length and consist of 10 open reading frames (ORFs). The genotypes of PRRSV-1 and PRRSV-2 are markedly different based on the full-length genomes, which share only approximately 60% similarity at the nucleotide level^2^.

PRRSV is recognized for its high genetic variation. Presently, PRRSV-1 and PRRSV-2 have continuously evolved into 3 subtypes and 9 lineages, respectively^3,4^. PRRSV-1 and PRRSV-2 have independently evolved in the European and North American (NA) continents. However, in Asia, the co-existence of both types has been increasingly evident in several countries, including Thailand, China, and Korea^5–7^. Additionally, variants of PRRSV-2 endemically present in Asia are genetically related to HP-PRRSV lineage 8.7/HP-PRRSV^8–10^.

Several PRRSV modified-live vaccines (MLV) against PRRSV-1 and PRRSV-2 have been commercially available and licensed in several countries worldwide depending on circulating virus genotypes. The use of PRRSV MLV depends on PRRSV genotype circulating in that region. However, questions have been raised as to what types of MLV should be used in the co-presence of PRRSV-1 and PRRSV-2. The criteria for vaccine selection should include the induction of the cell-mediated immunity (CMI) and the protection against PRRSV infection, especially against genotypes and isolates that are circulating in the affected region. Therefore, the present study was conducted to investigate CMI, IL-10, and protective efficacy of commercial PRRSV-1 and PRRSV-2 MLVs against co-challenge with PRRSV-1 and PRRSV-2 (HP-PRRSV). Our results revealed that vaccination with PRRSV MLVs, regardless of vaccine genotype, provide partial cross-protection against PRRSV infection. Additionally, this approach provided novel information regarding the vaccine selection for use in the presence of co-existence of both PRRSV genotypes.

## Materials and Methods

### Ethical statement for experimental procedures

All animal procedures were conducted in accordance with the Guide for the Care and Use of Laboratory Animals of the National Research Council of Thailand according to protocols reviewed and approved by the Chulalongkorn University Animal Care and Use Committee (protocol number 1731047).

Seventy, 21-day-old pigs were procured from a PRRS-free herd. Upon arrival, pigs were randomly allocated based on the stratification of weight into 7 treatment groups consisting of NonVac, Porcilis, Amervac, Fostera, Ingelvac MLV, Ingelvac ATP and Prime Pac (Table 1). Following a week of acclimatization, pigs were vaccinated with PRRS MLVs. NonVac was left unvaccinated. Porcilis and Amervac were vaccinated with Porcilis PRRS (PRRSV-1, MSD Animal Health, Boxmeer, the Netherlands) and Amervac PRRS (PRRSV-1, Laboratorios Hipra, Girona, Spain), respectively. Fostera, Ingelvac MLV, Ingelvac ATP and Prime Pac were vaccinated with Fostera PRRS (PRRSV-2, Zoetis, Troy Hills, USA), Ingelvac PRRS MLV (PRRSV-2, Boehringer Ingelheim, Rhein, Germany), Ingelvac PRRS ATP (PRRSV-2, Boehringer Ingelheim, Rhein, Germany) and Prime Pac PRRS (PRRSV-2, MSD Animal Health, Boxmeer, the Netherlands), respectively. Dosages and routes of administration were in accordance with manufacturers’ instructions. Blood samples were collected at 0, 7, 14, 21, 28, 35 days post-vaccination (DPV). Peripheral blood mononuclear cells (PBMC) were isolated and assayed for lymphocyte proliferative response. IFN-γ and IL-10 were measured using flowcytometry, and ELISPOT or ELISA. At 35 DPV, all pigs were inoculated intranasally with PRRSV. Each pig received 2 ml (1 ml/nostril) of each PRRSV-1 (AN06EU4204) and PRRSV-2 (FDT10US23) at 10^5.4^ TCID_50_/ml and 10^5.2^ TCID_50_/ml, respectively. Sera were collected at 0, 3, 5 and 7 days post-challenge (DPC) and quantitatively assayed for PRRSV RNA using qPCR. All pigs were necropsied at 7 DPC. The severity of PRRSV-induced pneumonic lung lesion was scored^11^. Lung tissues were collected for histopathological examination and immunohistochemistry (IHC).

**Table 1:** Experimental design. The pigs were allocated into seven treatment groups and vaccinated with six different PRRSV MLVs. The NonVac group was kept as unvaccinated control group.

Treatment groups	No. of pigs	Vaccination	Vaccines	Vaccine genotype	Dosage and route of administration	Manufacturers
NonVac	10	No	—	—	—	—
Porcilis	10	Yes	Porcilis PRRS	PRRSV-1	2 ml, intramuscular	MSD Animal Health, The Netherlands
Amervac	10	Yes	Amervac PRRS	PRRSV-1	2 ml, intramuscular	Laboratorios Hipra, Spain
Fostera	10	Yes	Fostera PRRS	PRRSV-2	2 ml, intramuscular	Zoetis, USA
Ingelvac MLV	10	Yes	Ingelvac PRRS MLV	PRRSV-2	2 ml, intramuscular	Boehringer Ingelheim, Germany
Ingelvac ATP	10	Yes	Ingelvac PRRS ATP	PRRSV-2	2 ml, intramuscular	Boehringer Ingelheim, Germany
Prime Pac	10	Yes	Prime Pac PRRS	PRRSV-2	1 ml, intramuscular	MSD Animal Health, The Netherlands

### Virus isolates

Homologous and heterologous viruses were used as recall antigens in *in vitro* CMI and IL-10 assays. Homologous viruses refer to vaccine strains as previously described^12^. Heterologous viruses refer to AN06EU4204 and FDT10US23, which were Thai PRRSV-1 and PRRSV-2 (HP-PRRSV) isolates, respectively. AN06EU4204 and FDT10US23 are in Clade A, Subtype 1 and Lineage 8.7/HP-PRRSV, respectively, based on systematic classification previously described^3,4^. ORF5 gene sequences of AN06EU4204 and FDT10US23 are available in GenBank under accession numbers JQ040750 and JN255836, respectively. The nucleotide and amino acid similarities based on the ORF5 gene between these two isolates and PRRSV MLVs were summarized in Table 2.

**Table 2:** Nucleotide and amino acid similarities based on ORF5 gene between vaccine strains and Thai PRRSV isolates.

PRRSV (isolates)	Classification*	Nucleotide and amino acid similarities						
		Level of similarity	Porcilis^®^ PRRSV	Amervac^®^ PRRSV	Fostera^™^ PRRS	Ingelvac^®^ PRRS MLV	Ingelvac^®^ PRRS ATP	Prime Pac^®^ PRRS
PRRSV-1 (AN06EU4204)	Subtype I (Clade A)	Nucleotide	95.8%	92.7%	68.5%	68.3%	68.2%	67.9%
		Amino acid	92.0%	89.1%	60.9%	58.2%	55.5%	55.7%
PRRSV-2 (FDT10US23)	Lineage 8.7/HP-PRRSV	Nucleotide	68.8%	69.9%	94.0%	88.8%	90.2%	90.5%
		Amino acid	58.7%	59.8%	91.5%	87.5%	89.5%	91.8%

^*^International systematic classification was based on previously described, including PRRSV-1^3^ and PRRSV-2^4^, respectively.

### Isolation of peripheral blood mononuclear cells

Peripheral blood mononuclear cells (PBMC) were isolated from blood samples using gradient density centrifugation (Lymphosep, Biowest, Riverside, MO, USA) as previously described^13^. Isolated PBMC were resuspended in 1 ml complete media (RPMI-1640 media supplemented with 10% fetal bovine serum (FBS), 2 mM L-glutamine, and 50 μg/ml gentamicin). The viability of PBMC were determined by Trypan blue (Sigma-Aldrich, St. Louis, MO, USA) staining and more than 90% viability was used for lymphocyte proliferation assay, lymphocytes producing either IL-10 or IFN-γ, IFN-γ ELISPOT assay, and *in vitro* stimulation for IL-10 detection as described below.

### Lymphocyte proliferation assay

The lymphocyte proliferation assay assesses cell proliferation using membrane-bound 5-(and-6)-carboxyfluorescein diacetate, succinimidyl ester (CFSE, Molecular Probes, Eugene, OR, USA) and cell surface markers using flow cytometry. Briefly, 1 × 10^7^ cells/ml PBMC were incubated with CFSE at 37 °C for 10 min. After washing, CFSE-stained PBMC at 1 × 10^6^ cells were seeded into 96-well plate and co-cultured with MARC-145 cell lysate (mock suspension), PHA (10 μg/ml, Sigma-Aldrich, St. Louis, MO, USA), homologous and heterologous PRRSV at 0.01 multiplicity of infection (MOI). Following 5-day incubation, PBMC were stained with mouse anti-porcine CD4-FITC antibody (clone 74-12-14, SouthernBiotech, Birmingham, AL, USA) and mouse anti-porcine CD8-SPRD antibody (clone 76-2-11, SouthernBiotech, Birmingham, AL, USA). After washing, PBMC were suspended in 2% paraformaldehyde. The proliferation of T lymphocyte populations was measured using flow cytometry analysis (Beckman FC550, Beckman Coulter, Brea, CA, USA) with CXP software. The relative proliferative indices (PI) were calculated by using the percentage of proliferating cells in the virus stimulated well divided by the percentage of proliferating cells in the mock suspension well.

### Lymphocytes producing either IL-10 or IFN-γ

The percentage of PRRSV-specific lymphocytes producing either IL-10 or IFN-γ after *in vitro* stimulation with homologous or heterologous PRRSV were evaluated using a method previously described^13^. Briefly, 1 × 10^6^ PBMC were seeded into a 96-well plate containing mock suspension, PMA (25 ng/ml)/ionomycin (1 μM) (Sigma-Aldrich, St. Louis, MO, USA), and homologous and heterologous PRRSV at 0.01 MOI, and incubated for 96 hours. Following incubation, protein transport inhibitor (BD GolgiStop, BD Biosciences, San Jose, CA, USA) was added 12 hours prior to cell harvesting and labeled PBMC were stained with mouse anti-porcine CD4-FITC antibody (clone 74-12-4, SouthernBiotech, Birmingham, AL, USA) and mouse anti-porcine CD8-SPRD antibody (clone 76-2-11, SouthernBiotech, Birmingham, AL, USA). Cells were subsequently fixed with fixation buffer (Leucoperm reagent A, Bio-Rad Laboratories, Hercules, CA, USA) for 15 min, washed and then separately incubated with either mouse anti-porcine IFN-γ-biotin antibody (clone P2C11, BD Pharmingen, San Jose, CA, USA) or mouse anti-porcine IL-10-biotin antibody (clone 945 A 1A9 26C2, Invitrogen, Carlsbad, CA, USA) in Leucoperm reagent B (Bio-rad Laboratories, Hercules, CA, USA). Subsequently, streptavidin-PE-Cy7 (Thermo Fisher Scientific, Waltham, MA, USA) were added and incubated for 30 min at 4 °C. After washing, stained cells were suspended in 2% paraformaldehyde and analyzed by flow cytometer (Beckman FC550, Beckman Coulter, Brea, CA, USA) with CXP software. The results are based on lymphocyte gating on a forward scatter versus side scatter graph after acquiring at least 20,000 cell events.

### Enzyme-linked immunospot (ELISPOT) assay

The numbers of PRRSV-specific interferon-γ-producing cells (IFN-γ-PC) were determined using ELISPOT kit (R&D Systems, Minneapolis, MN, USA). Briefly, 2 × 10^5^ PBMC were stimulated with either homologous or heterologous PRRSV at 0.01 MOI or PHA (10 μg/ml, Sigma-Aldrich, St. Louis, MO, USA) for 20 hours at 37 °C in 5% CO_2_. Spots were counted by an automated ELISPOT Reader (AID ELISPOT Reader, AID GmbH, Strassberg, Germany). PRRSV-specific IFN-γ-PC was expressed as spot forming colonies per million of PBMCs in each well.

### Quantification of porcine interleukin-10

Porcine interleukin-10 (IL-10) concentration was quantified in the supernatant of stimulated PBMC (2 × 10^6^ cells/well) cultured *in vitro* for 20 hours with homologous and heterologous PRRSV (0.01 of MOI) or PHA (10 μg/ml, Sigma-Aldrich, St. Louis, MO, USA) using the porcine ELISA IL-10 kit (R&D Systems, Minneapolis, MN, USA) according to manufacturer’s instruction.

### Quantification of PRRSV RNA

The PRRSV RNA in serum was evaluated by quantitative PCR (qPCR) after PRRSV challenge. The primers specific for the ORF5 gene of either PRRSV-1 or PRRSV-2 and detection conditions were described previously^12^. In brief, total RNA was extracted using NucleoSpin RNA Virus extraction kit (Macherey-Nagel, Duren, Germany) in accordance with manufacturer’s instructions. The quality of RNA was measured using spectrophotometer (Colibri, Titertek-Berthold, Pforzheim, Germany), and converted to cDNA. All cDNA was used for quantitative PCR (qPCR). PRRSV RNA was quantified using ABI PRISM 7500 Real time PCR platform (Applied Biosystem, CA, USA). Primers specific for the ORF5 gene of either PRRSV-1 or PRRSV 2 were used. Each qPCR reaction contained 0.1 μg of cDNA, 0.2 μM of each primers, 1x Eva Green real-time-PCR master mix E4 (GeneOn GmbH, Ludwigshafen, Germany), and deionized water to yield a 20 ul final volume. The thermal profile for qPCR was 94 °C for 4 min, followed by 35 cycles of 94 °C for 45 s, 55 °C for 45 s, and fluorescence acquisition at 72 °C for 45 s. pGEM-T Easy Vector (Promega, WI, USA) containing an inserted ORF5 gene of each PRRSV was used to construct plasmid standards. A standard curve was generated using serial diluted plasmid standards of 10^0^–10^7^ copies/μl. Copy number of the PRRSV RNA was calculated using standard curve method.

### Pathological examination and immunohistochemistry

All pigs were necropsied at 7 DPC. PRRSV-induced pneumonic lung lesions were macroscopically and microscopically evaluated as previously described^11^. For the macroscopic lung lesion score, each lung lobe was assigned a number to reflex the approximate percentage of the volume of the entire lung and the percentage volume form each lobe added to the entire lung score (ranged from 0 to 100% of the affected lung). For the microscopic lung lesion score, lung sections were blind. Histopathological changed were examined and an estimated score of the severity of the interstitial pneumonia was given as follows: 0 = no microscopic lesions; 1 = mild interstitial pneumonia; 2 = moderate multifocal interstitial pneumonia; 3 = moderate diffuse interstitial pneumonia; and 4 = severe interstitial pneumonia. The mean values of microscopic score of each group were calculated.

Immunohistochemistry was performed using monoclonal antibodies (MAbs) A35 and JP24, which recognized PRRSV-1 and PRRSV-2 antigens, respectively (kindly provided by Dr. Erwin van den Born, the Netherlands). Tissues were processed and placed on Superfrost Plus slides (Thermo Fisher Scientific, Waltham, MA, USA). Sections were deparaffinized, rehydrated using an alcohol gradient and air-dried. All slides were treated with proteinase K (Thermo Fisher Scientific, Waltham, MA, USA) in PBS for 30 min. Endogenous alkaline phosphatase was quenched with 0.3% hydrogen peroxide for 5 min. All slides were then incubated with BSA for 30 min. The slides were separately incubated with monoclonal antibodies overnight at 4 °C in a humidified chamber. After washing, PRRSV antigen was visualized by binding with secondary antibody conjugated with horseradish peroxidase conjugated (HRP)-labeled polymer followed by immersion in peroxidase (Agilent, Santa Clara, CA, USA). Slides were counterstained with Meyer’s hematoxylin, dehydrated through graded concentrations of ethanol and xylene, and then mounted. Lung tissues from pigs in the unvaccinated unchallenged group served as negative controls. To obtain quantitative data, slides were analyzed with the NIH Image J 1.50i Program (http://rsb.info.nih.gov/ij). In each slide, 10 fields were randomly selected, and the number of positive cells per unit area (0.95 mm^2^) was determined as previously described^14,15^. The mean values were calculated.

### Statistical analysis

The data from repeated measurements were analyzed using multivariate analysis of variance (ANOVA). Continuous variables were analyzed by ANOVA to determine the presence of significant differences between treatment groups for each day. If the *p*-value for the ANOVA was <0.05, the differences between treatment groups were evaluated by pairwise comparisons using least significant differences at the *p* < 0.05 significance level.

## Results

### Lymphocyte proliferation response using CFSE

**Figure 1:**
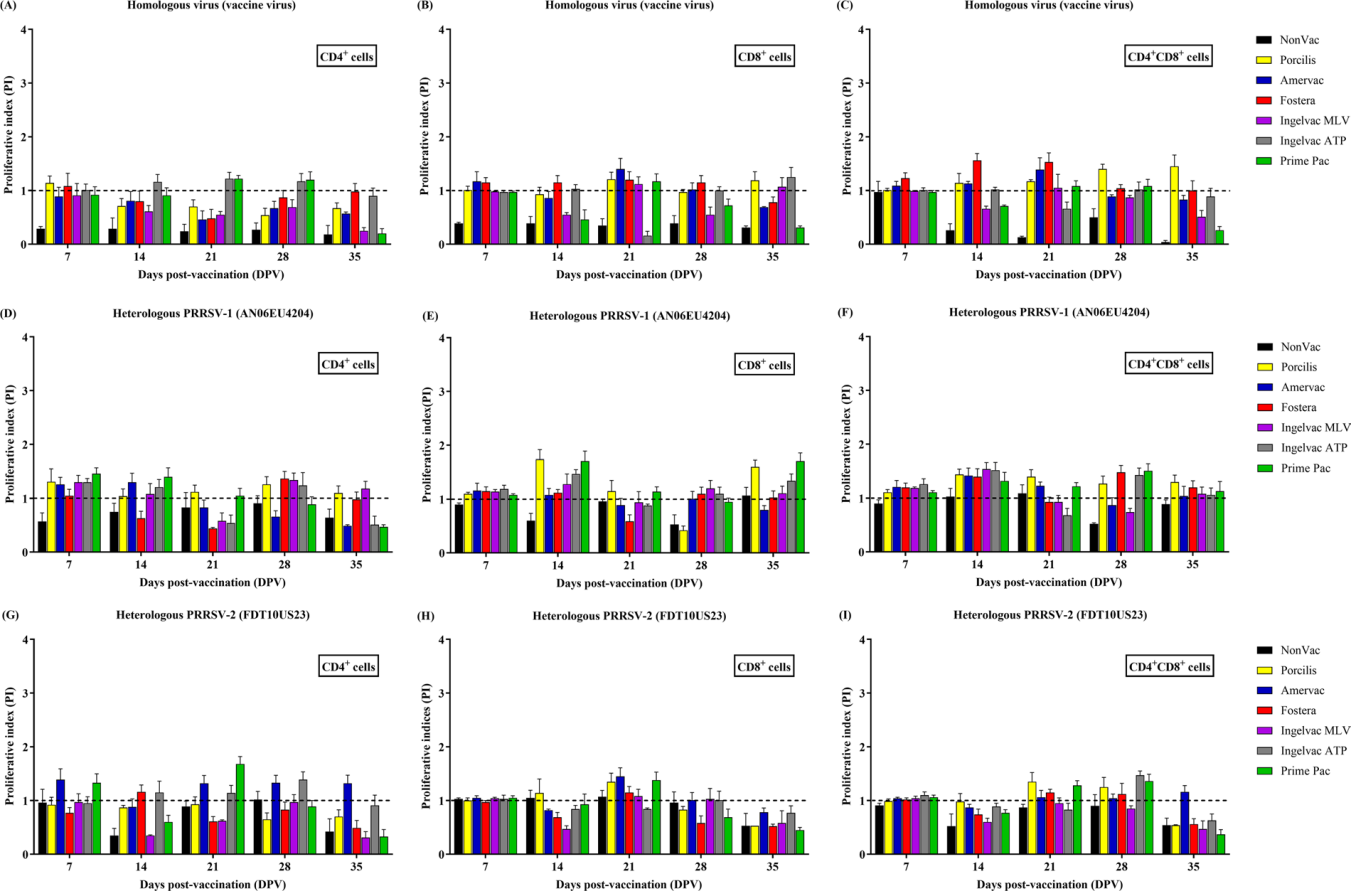
Lymphocyte proliferative index (PI) following vaccination. (**A**–**C**) Homologous virus (vaccine strain), (**D**–**F**) heterologous PRRSV-1 (AN06EU4204), and (**G**–**I**) heterologous PRRSV-2 (FDT10US23), respectively. The lymphocyte populations were identified by flow cytometry using CFSE and cell surfaces staining, including CD4^+^ cells (**A,D**,**G**), CD8^+^ cells (**B,E**,**H**), and CD4^+^CD8^+^ cells (**C,F**,**I**), respectively. Values are expressed as mean±SEM. Dash lines indicate the cut-off level.

Upon *in vitro* stimulation with either homologous or heterologous PRRSV, all vaccination groups, regardless of vaccine genotype, had relatively low lymphocyte proliferative indices following vaccination. A significantly increased response was not observed in any vaccination group, and the responses were not different among all of the vaccination groups (Fig. 1).

### Lymphocyte populations producing IL-10

**Figure 2:**
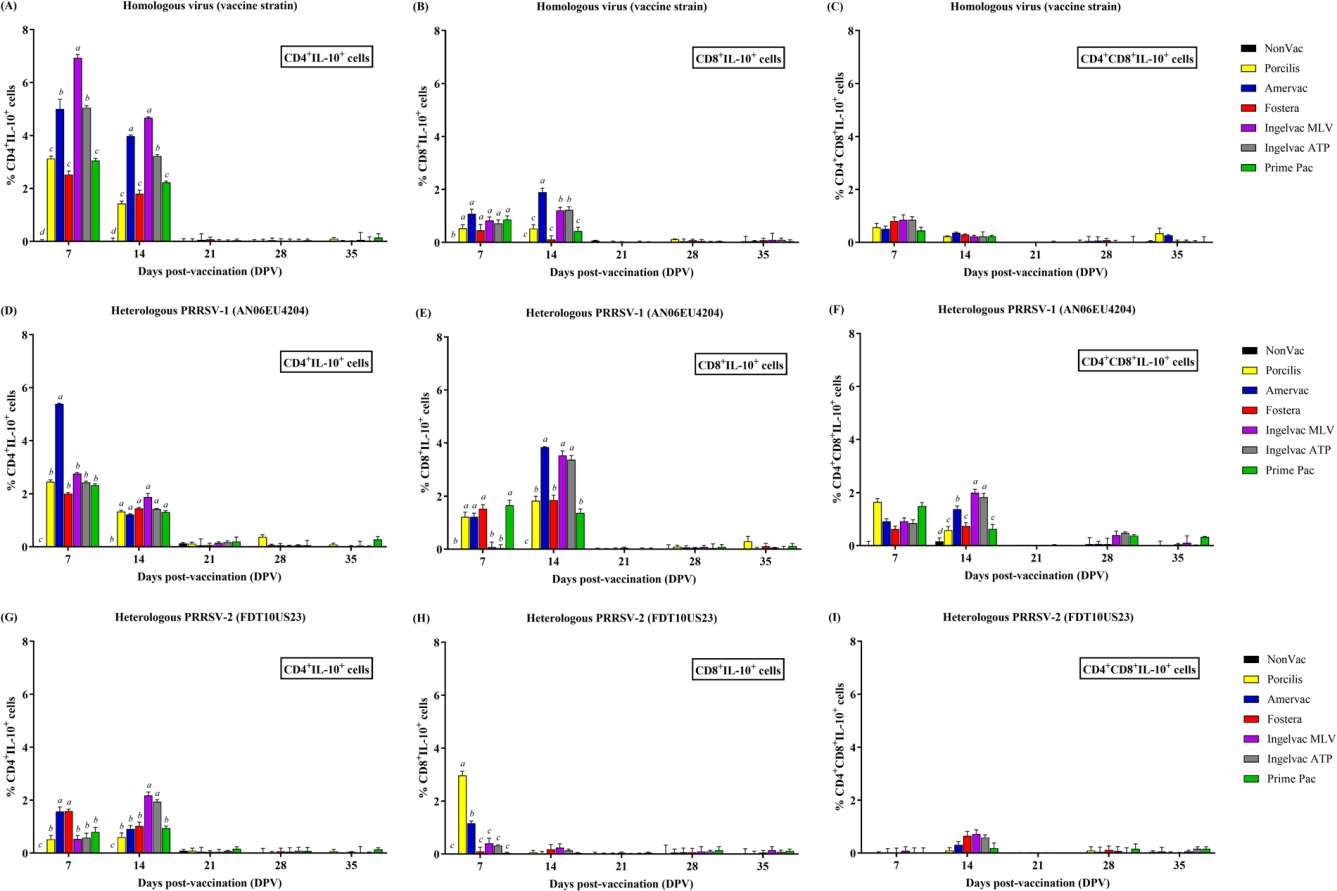
Lymphocyte populations producing IL-10 following vaccination. (**A**–**C**) Homologous virus (vaccine strain), (**D**–**F**) heterologous PRRSV-1 (AN06EU4204), and (**G**–**I**) heterologous PRRSV-2 (FDT10US23), respectively. The lymphocyte populations producing IL-10 were identified by flow cytometry using cell surfaces and intracellular IL-10 staining, including CD4^+^IL-10^+^ cells (**A,D**,**G**), CD8^+^IL-10^+^ cells (**B,E**,**H**), and CD4^+^CD8^+^IL-10^+^ cells (**C,F**,**I**), respectively. Values are expressed as mean±SEM. Results were compared using two-way ANOVA multiple comparison test. Lowercase letters represent significant differences between treatment groups (*p* < *0.05*) at each day post vaccination.

Following vaccination, lymphocyte populations producing IL-10 (L-IL-10) were detected in all vaccination groups at 7 and 14 DPV, regardless of vaccine genotype (Fig. 2). The percentage of L-IL-10 declined to a nondetectable level from 21 to 35 DPV. L-IL-10 was mainly produced by CD4^+^ cells. At 7 DPV, the Ingelvac MLV group had the highest amount of CD4^+^IL-10^+^ cells as compared to the PRRSV-1 or PRRSV-2 MLV vaccination and NonVac groups (Fig. 2A). CD4^+^IL-10^+^ cells in the Amervac, Ingelvac MLV and Ingelvac ATP groups were significantly higher than those in the other vaccination groups at 14 DPV. The Porcilis, Fostera and Prime Pac groups had the lowest amount of CD4^+^IL-10^+^ cells as compared to other PRRSV-1 and PRRSV-2 MLV vaccination groups (*p* < 0.05) at both 7 and 14 DPV.

Similar to CD4^+^IL-10^+^ cells, all vaccination groups had significantly more CD8^+^IL-10^+^ cells as compared to the NonVac group (Fig. 2B). Although there was no difference in CD8^+^IL-10^+^ cells among vaccination groups at 7 DPV, the Amervac, Ingelvac MLV, and Ingelvac ATP groups had significantly more CD8^+^IL-10^+^ cells than did the Porcilis, Fostera and Prime Pac groups at 14 DPV. Additionally, the Amervac group had the highest amount of CD8^+^IL-10^+^ cells as compared to other vaccination groups as 14 DPV. All vaccination groups had relatively more CD4^+^CD8^+^IL-10^+^ cells than did the NonVac group, and CD4^+^CD8^+^IL-10^+^ cell numbers were not different between the vaccination groups (Fig. 2C).

Similar to homologous virus stimulation, L-IL-10 was detected in all vaccination groups at 7 and 14 DPV after stimulation with PRRSV-1 (AN06EU4204) and was not detected from 21 to 35 DPV (Fig. 2D–F). All vaccination groups had higher amounts of CD4^+^IL-10^+^ cells than did the NonVac group at 7 and 14 DPV (Fig. 2D). The Amervac group had the highest amount of CD4^+^IL-10^+^ cells as compared to the PRRSV-1 and PRRSV-2 MLV vaccination groups at 7 DPV (*p* < 0.05). Meanwhile, at 14 DPV, there were no differences in CD4^+^IL-10^+^ cells among all of the vaccination groups.

The Porcilis, Amervac, Fostera and Prime Pac groups had significantly more CD8^+^IL-10^+^ cells than the NonVac, Ingelvac MLV and Ingelvac ATP groups at 7 DPV (Fig. 2E). However, at 14 DPV, all vaccination groups had significantly more CD8^+^IL-10^+^ cells than the NonVac group. The Amervac, Ingelvac MLV and Ingelvac ATP groups had significantly more CD8^+^IL-10^+^ cells than the Porcilis, Fostera and Prime Pac groups (*p* < 0.05).

All vaccination groups had more CD4^+^CD8^+^IL-10^+^ cells than the NonVac group at 7 and 14 DPV (Fig. 2F). At 7 DPV, no significant differences were detected in the amount of CD4^+^CD8^+^IL-10^+^ among all of the vaccination groups. In contrast, the Ingelvac MLV and Ingelvac ATP groups had more CD4^+^CD8^+^IL-10^+^ cells than the other vaccination groups (*p* < 0.05) at 14 DPV.

Following heterologous stimulation with PRRSV-2 (FDT10US23), L-IL-10 was detected at 7 and 14 DPV but not at 21 to 35 DPV (Fig. 2G–I). At both 7 and 14 DPV, all vaccination groups had higher levels than the NonVac group. At 7 DPV, the Amervac and Fostera groups had significantly more CD4^+^IL-10^+^ cells than the Porcilis, Ingelvac MLV, Ingelvac ATP and Prime Pac groups (*p* < 0.05). However, at 14 DPC, the amount of CD4^+^IL-10^+^ cells was the highest in the Ingelvac MLV and Ingelvac ATP groups as compared to the other vaccination groups (Fig. 2G). In contrast, CD8^+^IL-10^+^ cells were only detected at 7 DPV (Fig. 2H) in all vaccination groups, and the Porcilis group had more CD8^+^IL-10^+^ cells than the other groups. There were no differences in CD4^+^CD8^+^IL-10^+^ cells among all of the vaccination groups after stimulation with PRRSV-2 (Fig. 2I).

### Lymphocyte Populations Producing IFN-γ

**Figure 3:**
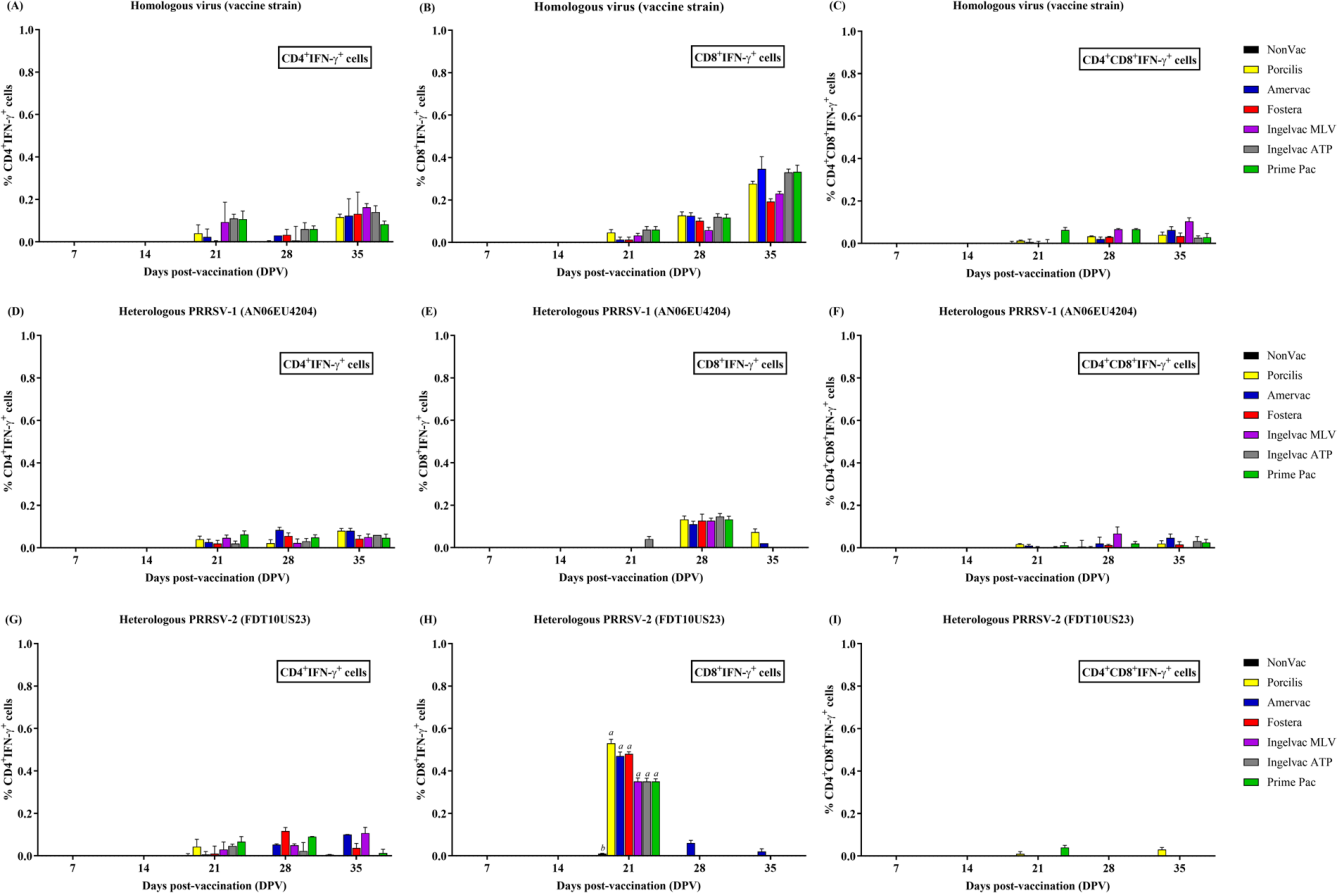
Lymphocyte populations producing IFN-γ following vaccination. (**A**–**C**) homologous virus (vaccine strain), (**D**–**F**) heterologous PRRSV-1 (AN06EU4204), and (**G**–**I**) heterologous PRRSV-2 (FDT10US23), respectively. The lymphocyte populations producing IFN-γ were identified by flow cytometry using cell surfaces and intracellular IFN-γ staining, including CD4^+^IFN-γ^+^ cells (**A,D**,**G**), CD8^+^IFN-γ^+^ cells (**B,E**,**H**), and CD4^+^CD8^+^IFN-γ^+^ cells (**C,F**,**I**), respectively. Values are expressed as mean±SEM. Results were compared using two-way ANOVA multiple comparison test. Lowercase letters represent significant differences between treatment groups (*p* < *0.05*) at each day post vaccination.

Lymphocyte populations producing IFN-γ (L-IFN-γ) were detected after stimulation with either homologous or heterologous PRRSV as early as 21 DPV at levels less than 1% in all vaccination groups and showed no statistical differences between vaccination groups. Soon after detection, L-IFN-γ gradually increased until 35 DPV (Fig. 3). The lymphocyte population response was toward both CD4^+^ and CD8^+^. Immediately after homologous stimulation, all vaccination groups had relatively more CD4^+^IFN-γ^+^ and CD8^+^IFN-γ^+^ cells than the NonVac group but showed no differences thereafter (Fig. 3A–C). Similar to homologous stimulation, all vaccination groups had relatively more CD4^+^IFN-γ^+^ and CD8^+^IFN-γ^+^ cells after stimulation with heterologous PRRSV-1 (AN06EU4204) than the NonVac group but showed no difference among vaccination groups (Fig. 3D–F). However, after heterologous PRRSV-2 (FDT10US23) stimulation, CD8^+^IFN-γ^+^ cells in all vaccination groups were detected only at 21 DPV and were significantly greater in number than in the NonVac group (Fig. 3H).

### The number of PRRSV-specific IFN-γ-PC

**Figure 4:**
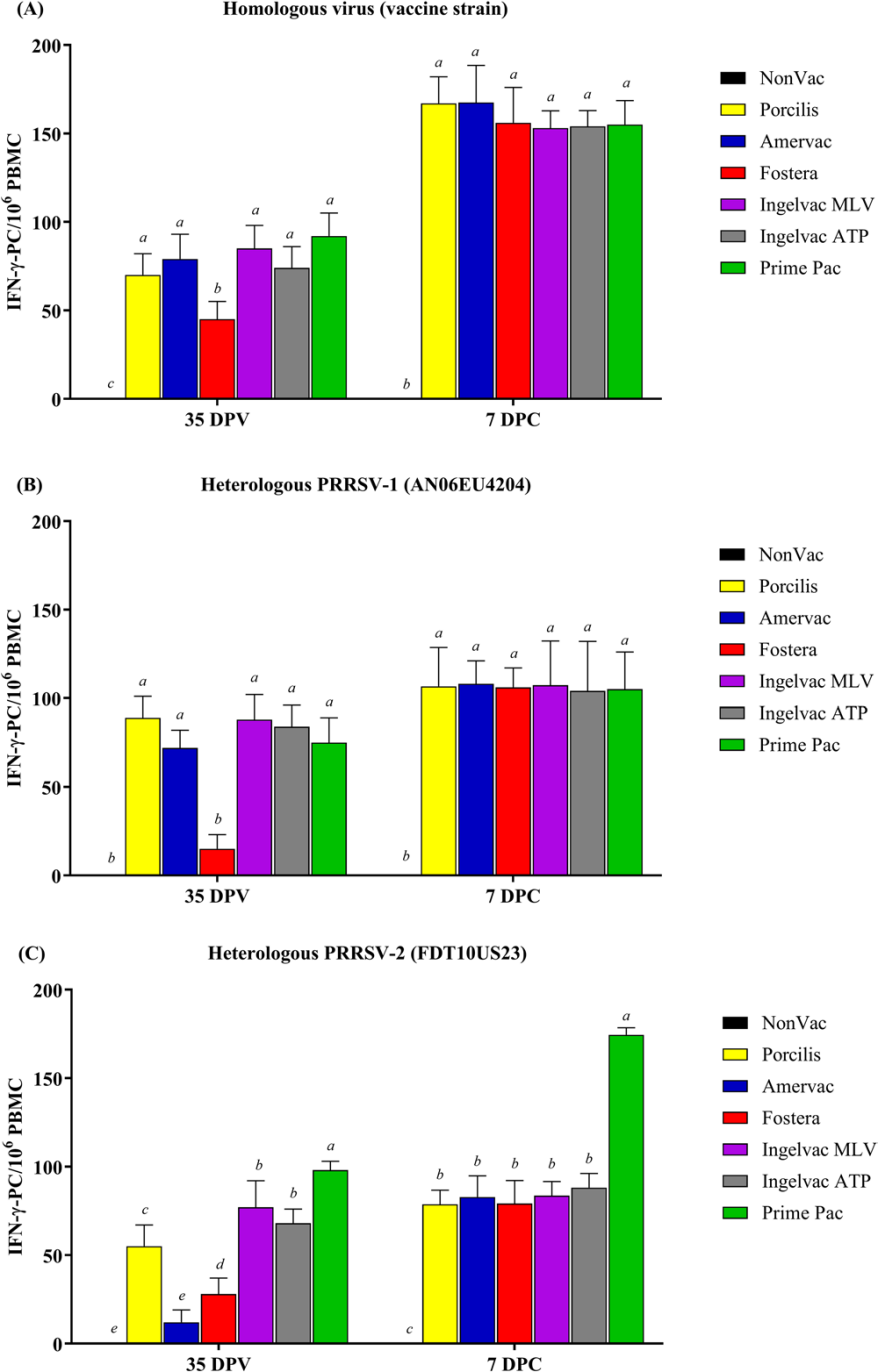
Evaluation of PRRSV-specific IFN-γ-PC following vaccination and at 7 days post-challenge (DPC) using *in vitro* stimulation. (**A**) Homologous virus (vaccine strain), (**B**) heterologous PRRSV-1 (AN06EU4204), and (**C**) heterologous PRRSV-2 (FDT10US23), respectively. Values are expressed as mean±SEM. Results were compared using two-way ANOVA multiple comparison test. Lowercase letters represent significant differences between treatment groups (*p* < *0.05*) at each day post vaccination.

Regardless of homologous or heterologous stimulation, IFN-γ-PC of all vaccination groups were first detected at 35 DPV (Fig. 4). After homologous stimulation, all vaccination groups had significantly more IFN-γ-PC than the NonVac group at 35 DPV and 7 DPC. The Fostera group had significantly fewer IFN-γ-PC than the other vaccination groups at 35 DPV (Fig. 4A). After heterologous PRRSV-1 (AN06EU4204) stimulation, all vaccination groups had significantly more IFN-γ-PC than NonVac group at 35 DPV and 7 DPC. The Fostera group had significantly fewer IFN-γ-PC than the other vaccination groups at 35 DPV (Fig. 4B). After heterologous PRRSV-2 (FDT10US23) stimulation, IFN-γ-PC numbers were lower in all vaccination groups than after homologous stimulation with the exception of the Prime Pac group, which had significantly more IFN-γ-PC than the other groups at 35 DPV and 7 DPC. IFN-γ-PC were less abundant in Amervac the group than the other vaccination groups and were not different from those in the NonVac group at 35 DPV (Fig. 4C).

### Porcine IL-10 production

**Figure 5:**
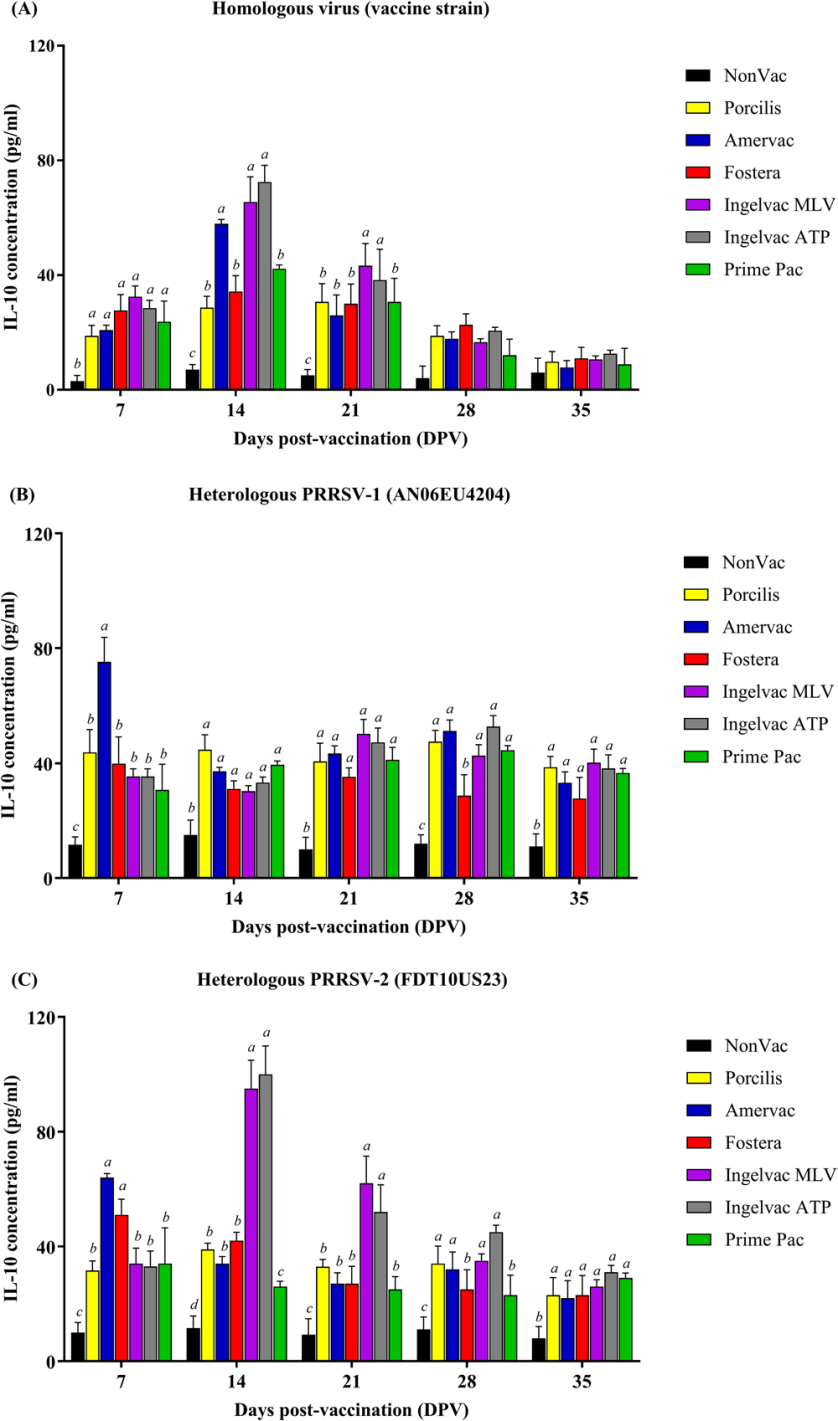
Quantification of porcine IL-10 in supernatant of stimulated PBMC following vaccination. (**A**) Homologous virus (vaccine strain), (**B**) heterologous PRRSV-1 (AN06EU4204), and (**C**) heterologous PRRSV-2 (FDT10US23), respectively. Values are expressed as mean ± SEM. Results were compared using two-way ANOVA multiple comparison test. Lowercase letters represent significant differences between treatment groups (*p* < *0.05*) at each day post vaccination.

After homologous stimulation, IL-10 levels in all vaccination groups increased and were significantly higher than that in the NonVac group at 7 DPV (Fig. 5A). IL-10 levels of all vaccination groups peaked at 14 DPV and gradually decreased until they were similar to that of the NonVac group at 35 DPV. At 7 DPV, no differences were detected in IL-10 levels between the vaccination groups. The Amervac, Ingelvac MLV and Ingelvac ATP groups had significantly higher IL-10 levels at 14 DPV than the Porcilis, Fostera and Prime Pac groups. The IL-10 levels of the Ingelvac MLV and Ingelvac ATP groups remained significantly higher at 21 DPV compared to those of the other vaccination groups. No differences were detected in IL-10 among all of the vaccination groups at 28 or 35 DPV.

After heterologous PRRSV-1 (AN06EU4204) stimulation, IL-10 levels were significantly higher in all vaccination groups than that in the NonVac group (Fig. 5B). The Amervac group had a significantly higher IL-10 level than did the other vaccination groups at 7 DPV. However, no differences were detected in IL-10 in all vaccination groups from 14 to 28 DPV, except for the Fostera group. The IL-10 level was significantly lower in the Fostera group on 28 DPV than those in the other vaccination groups.

After heterologous PRRSV-2 (FDT10US23) stimulation, all vaccination groups had significantly higher IL-10 levels than the NonVac group (Fig. 5C). The Amervac and Fostera groups had significantly higher IL-10 levels at 7 DPV as compared to those of the other vaccination groups. The IL-10 levels in all vaccination groups, except for the Ingelvac MLV and Ingelvac ATP groups, continuously decreased from 7 to 35 DPC. The Ingelvac MLV and Ingelvac ATP groups had significantly higher IL-10 levels at 14 and 21 DPV as compared to those of the other vaccination groups. At 28 DPV, IL-10 levels were significantly lower in the Fostera and Prime Pac groups than in the other vaccination groups but were still significantly higher than that the NonVac group. No statistical differences were observed in IL-10 levels between vaccination groups at 35 DPV.

### PRRSV RNA in serum

Serum PRRSV RNA quantification after co-challenge was summarized in Table 3. Regardless of vaccine genotype, all vaccination groups had significantly (*p* < 0.05) lower levels of both PRRSV-1 and PRRSV-2 RNA as compared to those of the NonVac group at 3, 5 and 7 DPC. Although, the PRRSV-2 MLV vaccination groups had significantly lower PRRSV-1 RNA levels compared to those of the PRRSV-1 MLV vaccination groups at 3 and 7 DPC, no differences were observed at 5 DPC between the PRRSV-2 MLV vaccination and the Amervac groups. At 5 DPC, serum PRRSV-1 RNA increased in all PRRSV-2 MLV vaccination groups as compared to those at 3 DPC. In contrast, PRRSV-1 RNA levels were reduced in all PRRSV-1 MLV vaccination groups at 5 and 7 DPC as compared to those at 3 DPC. The reduction in serum PRRSV RNA was not genotype-related but was associated with the isolates used in MLV. PRRSV-1 RNA was lower in the Porcilis group than in the Amervac group at 5 and 7 DPC. Meanwhile, no differences were detected in PRRSV-1 RNA levels between the PRRS-2 MLV vaccination groups at 5 DPC.

**Table 3:** Results of PRRSV RNA in sera of non-vaccinated and vaccinated pigs following co-challenge with PRRSV-1 and PRRSV-2, lung lesion scores and immunohistochemistry at 7 days post-challenge (DPC).

		DPC*	Treatment groups						
			NonVac	Porcilis	Amervac	Fostera	Ingelvac MLV	Ingelvac ATP	Prime Pac
PRRSV RNA (1,000 copies/ml)	PRRSV-1	0	0.0 ± 0.0^*¥*^	0.0 ± 0.0	0.0 ± 0.0	0.0 ± 0.0	0.0 ± 0.0	0.0 ± 0.0	0.0 ± 0.0
		3	2.3 ± 0.2^*a*^	1.4 ± 0.3^*b*^	1.3 ± 0.2^*b*^	0.2 ± 0.0^*d*^	0.6 ± 0.1^*c*^	0.5 ± 0.2^*c*^	0.2 ± 0.0^*d*^
		5	2.7 ± 0.3^*a*^	0.4 ± 0.1^*c*^	0.8 ± 0.2^*b*^	0.9 ± 0.1^*b*^	0.8 ± 0.2^*b*^	0.9 ± 0.3^*b*^	1.1 ± 0.2^*b*^
		7	1.8 ± 0.2^*a*^	0.4 ± 0.1^*c*^	0.8 ± 0.2^*b*^	0.5 ± 0.2^*c*^	0.1 ± 0.0^*d*^	0.5 ± 0.1^*c*^	0.4 ± 0.1^*c*^
	PRRSV-2	0	0.0 ± 0.0	0.0 ± 0.0	0.0 ± 0.0	0.0 ± 0.0	0.0 ± 0.0	0.0 ± 0.0	0.0 ± 0.0
		3	2.3 ± 0.2^*a*^	1.4 ± 0.2^*b*^	1.7 ± 0.2^*b*^	1.5 ± 0.2^*b*^	1.2 ± 0.2^*b*^	1.4 ± 0.3^*b*^	1.4 ± 0.2^*b*^
		5	2.7 ± 0.2^*a*^	1.5 ± 0.3^*b*^	0.8 ± 0.3^*c*^	1.3 ± 0.3^*b*^	1.8 ± 0.3^*b*^	1.3 ± 0.3^*b*^	0.8 ± 0.2^*c*^
		7	2.9 ± 0.2^*a*^	1.0 ± 0.3^*b*^	0.5 ± 0.3^*c*^	1.3 ± 0.3^*b*^	1.1 ± 0.3^*b*^	1.1 ± 0.5^*b*^	0.7 ± 0.2^*c*^
Macroscopic lung scores		7	72.7 ± 8.8^*a*^	59.0 ± 4.4^*a*^	45.0 ± 5.7^*b*^	55.3 ± 5.5^*b*^	54.7 ± 1.7^*b*^	54.6 ± 6.4^*b*^	42.7 ± 4.6^*c*^
Microscopic lung scores		7	1.40 ± 0.08^*a*^	1.24 ± 0.06^*a*^	0.92 ± 0.08^*b*^	0.82 ± 0.08^*b*^	0.83 ± 0.08^*b*^	0.82 ± 0.08^*b*^	0.87 ± 0.08^*b*^
PRRSV-antigen score by IHC^§^	A35	7	15.2 ± 1.8^*a*^	6.0 ± 0.7^*b*^	3.2 ± 0.4^*c*^	4.7 ± 0.4^*c*^	4.2 ± 0.3^*c*^	4.4 ± 0.3^*c*^	3.5 ± 0.2^*c*^
	JP24	7	8.2 ± 1.4^*a*^	4.9 ± 0.4^*b*^	3.9 ± 0.5^*b*^	2.3 ± 0.3^*c*^	4.0 ± 0.4^*b*^	4.1 ± 0.5^*b*^	2.6 ± 0.2^*c*^

^*^Days post-challenge (DPC).

^§^Immunohistochemistry (IHC) using A35 and JP24, monoclonal antibodies specifically against PRRSV-1 and PRRSV-2 antigens, respectively.

^¥^Values are displayed in mean ± SEM. The different lowercase letters represent significant differences between treatment groups (*p* < 0.05) at each day.

The PRRSV-2 RNA results are similar to those of PRRSV-1 RNA. All vaccination groups had significantly lower PRRSV-2 RNA as compared to that of the NonVac group, regardless of the vaccine genotype. In addition, PRRSV RNA levels were not different between vaccination groups at 3 DPC. At 5 and 7 DPC, PRRSV-2 RNA levels remained similar levels compared to those at 3 DPC in all vaccination groups except the Amervac and Prime Pac groups, which had significantly lower serum PRRSV-2 RNA at 5 and 7 DPC as compared to the other vaccination groups.

### Pathological examination

For macroscopic lung lesion scores, the NonVac group had the highest PRRSV-induced pneumonic lung scores at 7 DPC (Table 3). In contrast, the lung lesion scores of all vaccination groups were significantly lower than that of the NonVac group regardless of genotype. The Porcilis group had the highest macroscopic lung lesion scores as compared to the other vaccination groups. The Prime Pac group had a significantly lower scores as compared to the other vaccination groups (Supplemetary information).

Microscopic lung lesions associated with PRRSV infection were characterized by thickened alveolar septa with increased numbers of interstitial macrophages and lymphocytes and by type II pneumocyte hyperplasia. The microscopic lung lesion score results were concordant with the macroscopic lung lesion score results. All vaccination groups, except the Porcilis group, had significantly lower microscopic lung lesion scores compared to the NonVac group (Table 3).

### Immunohistochemistry

Regardless of vaccine genotype, the mean number of PRRSV-positive cells was significantly (*p* < 0.05) lower in all vaccination groups as compared to the NonVac group using either A35 or JP24 MAbs (Table 3). The mean number of PRRSV-positive cells stained with A35 MAb in the Porcilis group was significantly (*p* < 0.05) higher than those in the other vaccination groups. No differences were detected in the mean number of PRRSV-positive cells between the Amervac group and the other PRRSV-2 MLV vaccination groups. In contrast, the Fostera and Prime Pac groups had significantly lower mean numbers of PRRSV-positive cells stained with JP24 MAb as compared to the Amervac, Ingelvac MLV and Ingelvac ATP groups (Supplementary information).

## Discussion

The present study was conducted to investigate CMI, IL-10 levels and protective efficacy of PRRSV-1 and PRRSV-2 MLVs against co-challenge with PRRSV-1 and PRRSV-2 (HP-PRRSV). Following PRRSV MLV vaccination, regardless of MLV genotype, the induction of CMI against PRRSV as measured by lymphocyte proliferative response and IFN-γ-PC against homologous stimulation was relatively delayed and low in magnitude. The response was observed beginning from 28–35 DPV. Additionally, the magnitude of the response was not different between vaccination groups. Although there was no difference in CMI, IL-10 was different between vaccination groups. Regardless of MLV genotype, increased IL-10 production was observed in all vaccination groups after vaccination. IL-10 levels were significantly higher in all vaccination groups at 7 DPV than in the unvaccinated control. The magnitude of the increase in IL-10 level is not genotype-related but rather is influenced by the virus isolate used to manufacture the vaccine. The Amervac, Ingelvac MLV and Ingelvac ATP groups had significantly higher IL-10 levels than the Porcilis, Fostera and Prime Pac groups. The Prime Pac group had the lowest IL-10 level. Following challenge, regardless of MLV genotype, all vaccinated pigs were partially protected against co-challenge with PRRSV-1 and PRRSV-2, as demonstrated by significantly reduced viremia against both genotypes, lung lesion scores and PRRSV antigens in lung tissues at 7 DPC as compared to the unvaccinated group, and the Prime Pac group demonstrated significantly greater reductions than the other vaccination groups. The results of reduced viremia and lung lesions suggest that protective efficacy against co-challenge with PRRSV-1 and PRRSV-2 (HP-PRRSV) is not genotype-related but rather is influenced by the virus isolate used to manufacture the vaccine. The results of the study suggest that all PRRSV MLVs are relatively similar in their protective efficacy against concurrent heterologous PRRSV-1 and PRRSV-2 (HP-PRRSV) challenge. The use of either genotype of PRRSV MLV to control PRRS in herds co-infected with both PRRSV genotypes would provide some level of protection against heterologous PRRSV infection. Other control strategies, including strict biosecurity, to prevent external PRRSV introduction will enhance a successful PRRSV control program.

Although CMI against either PRRSV MLV or field infection has been intensively studied^13,15–19^, no study has performed a comparative study between both MLV genotypes. The CMI results against the homologous virus in the present study demonstrated that all PRRSV MLVs induce relatively slow CMI responses as measured by lymphocyte proliferative response and the number of IFN-γ-PC, regardless of vaccine genotype. Based on the lymphocyte proliferative response, it was demonstrated that none of the PRRSV MLVs induced a detectable response until 35 DPV. The results of the CMI response analysis reported herein assessing CFSE-labeled lymphocyte proliferation are in agreement with those of previous reports showing that a PRRSV-specific CMI response appears late, approximately 4–6 weeks post-vaccination as determined by lymphocyte blastogenesis and other assays^13,20–22^. In contrast to the lymphocyte proliferative response, the CMI response, as measured by the enumeration of IFN-γ-PC, demonstrated that all MLV isolates induced a delay in the detectable level of response. After *in vitro* stimulation with homologous vaccine viruses, IFN-γ-PC were detected in pigs vaccinated with either PRRSV-1 or PRRSV-2 MLVs at 35 DPV and showed significantly higher numbers in the vaccination groups than in the NonVac group, albeit the numbers were relatively low. The number of IFN-γ-PC, however, increased rapidly by 7 DPC. The results of the delayed CMI response induced by MLV are in accordance with those of previous studies in which vaccination with either PRRSV-1 or PRRSV-2 MLV elicited a relatively slow CMI response^13,19,23^. The findings of the present study suggest that all commercial PRRSV MLVs induce a relative slow CMI response, regardless of vaccine genotype. Such responses are directed toward homologous stimulation.

It is noteworthy that the effective CMI response was directed toward the homologous response. The use of heterologous stimulation, either by PRRSV-1 or PRRSV-2, showed contrasting results to homologous stimulation. The heterologous response was somewhat unpredictable and unrelated to the genetic similarity between the vaccine and the challenge viruses. A previous study reported similar findings in that homologous stimulation upregulates IFN-γ-PC following vaccination, while heterologous virus stimulation showed varied IFN-γ-PC upregulation^13^. Heterologous stimulation with one virus was able to upregulate IFN-γ-PC as high as homologous stimulation, while another virus was not able to do so despite high genetic similarity. In the present study, the frequencies of IFN-γ-PC in PBMC varied after stimulation with heterologous recall viruses. Stimulation with either heterologous PRRSV-1 or PRRSV-2 induced low amounts of IFN-γ-PC in the Amervac and Fostera groups (Fig. 4B,C). In contrast, some vaccination groups, in particular the Prime Pac group, showed increased amounts of IFN-γ-PC after stimulation with heterologous PRRSV-2 (Fig. 4C). Our results are in accordance with those of previous studies suggesting that viral recognition is also directed against antigens of genetically divergent virus isolates regardless of the vaccine strain^13^. In addition, a cellular immune response such as IFN-γ-PC depends on the virus isolate used for *in vitro* stimulation, and different PRRSV isolates can interact differently to stimulate immune cells^23,24^.

Following vaccination, all vaccination groups had significantly higher IL-10 levels compared to the unvaccinated group (Fig. 5A–C). The IL-10 level decreased at 14 DPV and was not different between the MLV-vaccinated and unvaccinated groups at 21 DPV. It is noteworthy that while the IL-10 levels of most of the vaccination groups displayed a gradual declining trend after 7 DPV, the Amervac, Ingelvac MLV and Ingelvac ATP groups had increased levels of IL-10 until 14 DPV before showing a decline. Our result demonstrated that the patterns of IL-10 levels following PRRSV MLV vaccination were different regardless of genotype of MLV but were rather influenced by the PRRSV isolate used to manufacture the vaccine. The Amervac, Ingelvac MLV and Ingelvac ATP groups had significantly higher IL-10 levels than the Porcilis, Fostera and Prime Pac groups. These varying IL-10 levels among the vaccination groups may be due to the different virus isolates used in vaccine production or *in vitro* stimulation^23,25–28^. The differences in IL-10 levels among the PRRSV MLV vaccination groups are not surprising. Previous reports have demonstrated that PRRSV isolates vary in the degree of IL-10 production both *in vivo* and *in vitro*^17,29^. IL-10 induction by PRRSV might depend on the virus isolate used in the experiment^23,25–28^. Our findings support the conclusion that PRRSV MLVs, regardless of vaccine genotype, are able to induce IL-10 upregulation, thus resembling a natural PRRSV infection^30^. The level of IL-10 production depends on the virus isolate used in the vaccine^23^. This finding can be used as one of several criteria to select a vaccine to use for PRRSV control. A higher level of IL-10 can potentially induce more adverse effects following vaccination with PRRSV MLVs. A previous report demonstrated that following vaccination with Ingelvac MLV and Amervac, pigs had higher lung lesion scores compared to other vaccination groups^31^. This could be because IL-10 induction is higher in these groups than in other PRRSV MLV vaccination groups.

It is noteworthy that, regarding to the CMI response in the present study, we only investigated the dynamic change of immune cells against different PRRSV MLV vaccines using the lymphocyte proliferative assay. Our findings illustrated variations observed in the proliferative indices between PRRSV MLV vaccines. Although the CMI response as measured by the lymphocyte proliferative assay between vaccinated groups were difference, the degree of clinical protection after PRRSV infection was similar. The results suggested that CMI might not fit as immunological correlation for PRRSV protection. In agreement with our findings, previous studies found that the protection against PRRSV infection does not correlate with CMI response^32,33^. In addition, the dynamic change of immune cells seems not to correlate with other cytokines including IL-10. IL-10 is expressed by many cells of the adaptive immune system, including Th1, Th2 and Th17 cell subsets, Treg, CD8^+^ T cells and B cells^34–37^. It is also produced by cells of innate immune system including dendritic cells (DCs), macrophages, mast cells, natural killer (NK) cells, eosinophils and neutrophils^35^. The uncorrelated results could be due to IL-10 produced from these cells. Unfortunately, the defined immune cell subpopulations involved in the different CMI response between PRRSV MLV vaccines in the present study are not fully characterize due to the limitation of cell-specific antibodies. Additional studies to measure subpopulations of immune cells secreting cytokines against PRRSV MLV vaccines are needed for further investigation.

Genetic similarity between the vaccine and field virus is not a good indicator of the protective efficacy provided by a PRRSV MLV vaccine^38^. The protective efficacy of a PRRSV MLV is usually determined by the reduction in viremia and lung lesions following challenge with field viruses^39,40^. In the present study, vaccination with either PRRSV-1 or PRRSV-2 MLVs reduced the level of PRRSV viremia, lung lesions, both macroscopically and microscopically, and PRRSV antigen in the lung tissues of vaccinated pigs following co-challenge with heterologous PRRSV-1 and PRRSV-2 compared to the non-vaccinated control. Based on pneumonic lung lesions, all PRRSV MLVs provide some level of protection against co-infection with PRRSV-1 and PRRSV-2, regardless of vaccine genotype. The lung lesion scores and PRRSV antigens in lung tissues were significantly reduced in the vaccination groups compared to the unvaccinated group after challenge with heterologous viruses^15–18^. Our results are in agreement with those of a previous single challenge study that demonstrated partial cross-protection by PRRSV MLV^15,16,22,41–44^. On the other hand, our cross-protection results are in contrast with those of another previous dual-challenge study in which vaccination with PRRSV-1 MLV reduced only PRRSV-1 viremia and not PRRSV-2 viremia^45^. Pigs vaccinated with PRRSV-1 MLV showed no reductions in PRRSV-2 antigens in lung tissues. The discrepancy between these findings could be due to the virus isolate used in the two studies. It is possible that the differences are attributable to our challenge strain of PRRSV-2 having higher levels of virulence. To postulate, additional studies are needed.

## Conclusion

Based on the overall results of the present study, all commercially available PRRSV MLVs are capable of inducing relatively low and delayed CMI response. Differences in IL-10 responses post vaccination were noted between the different vaccines. Vaccination with PRRS MLVs will reduce viremia and lung lesions after heterologous PRRSV challenge regardless of vaccine genotype.

## Supplementary information

Supplementary information.
