# Supplementary material for: Cell-mediated immune response and protective efficacy of porcine reproductive and respiratory syndrome virus modified-live vaccines against co-challenge with PRRSV-1 and PRRSV-2
Source: Sci Rep. 2020 Feb 3;10:1649. doi: 10.1038/s41598-020-58626-y (PMC6997162; doi:10.1038/s41598-020-58626-y)
Supplement: Supplementary file 1 — Supplementary information. [file 41598_2020_58626_MOESM1_ESM.pdf]

Supplementary Information

Cell-mediated immune response and protective efficacy of porcine reproductive and respiratory syndrome virus modified-live vaccines against co-challenge with PRRSV-1 and PRRSV-2

Adthakorn Madapong, Kepalee Saeng-chuto, Alongkot Boonsoongnern, Angkana Tantituvanont and Dachrit Nilubol

Supplementary Figures

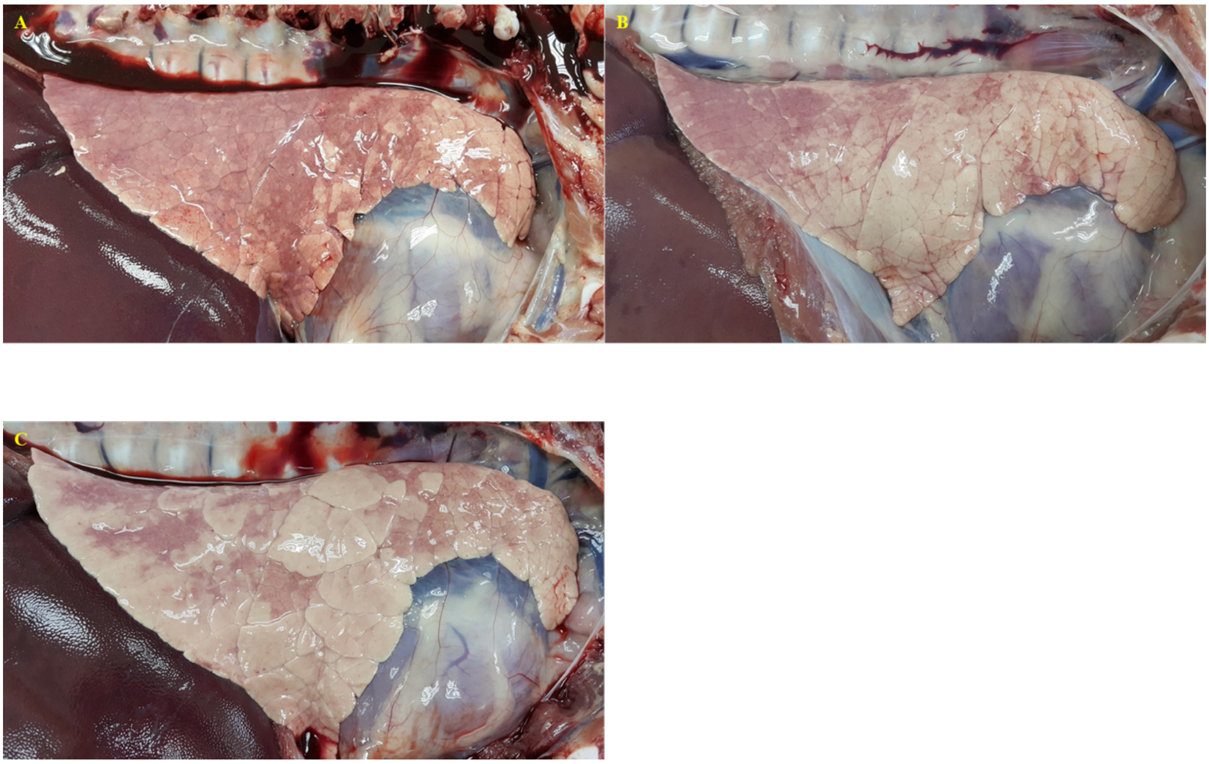

Supplementary Figure S1. Macroscopic lung lesion of the unvaccinated challenged (**A**), PRRSV-1 MLV, and PRRSV-2 MLV vaccinated challenged (**B** and **C**) pigs at 7 days post-challenge.

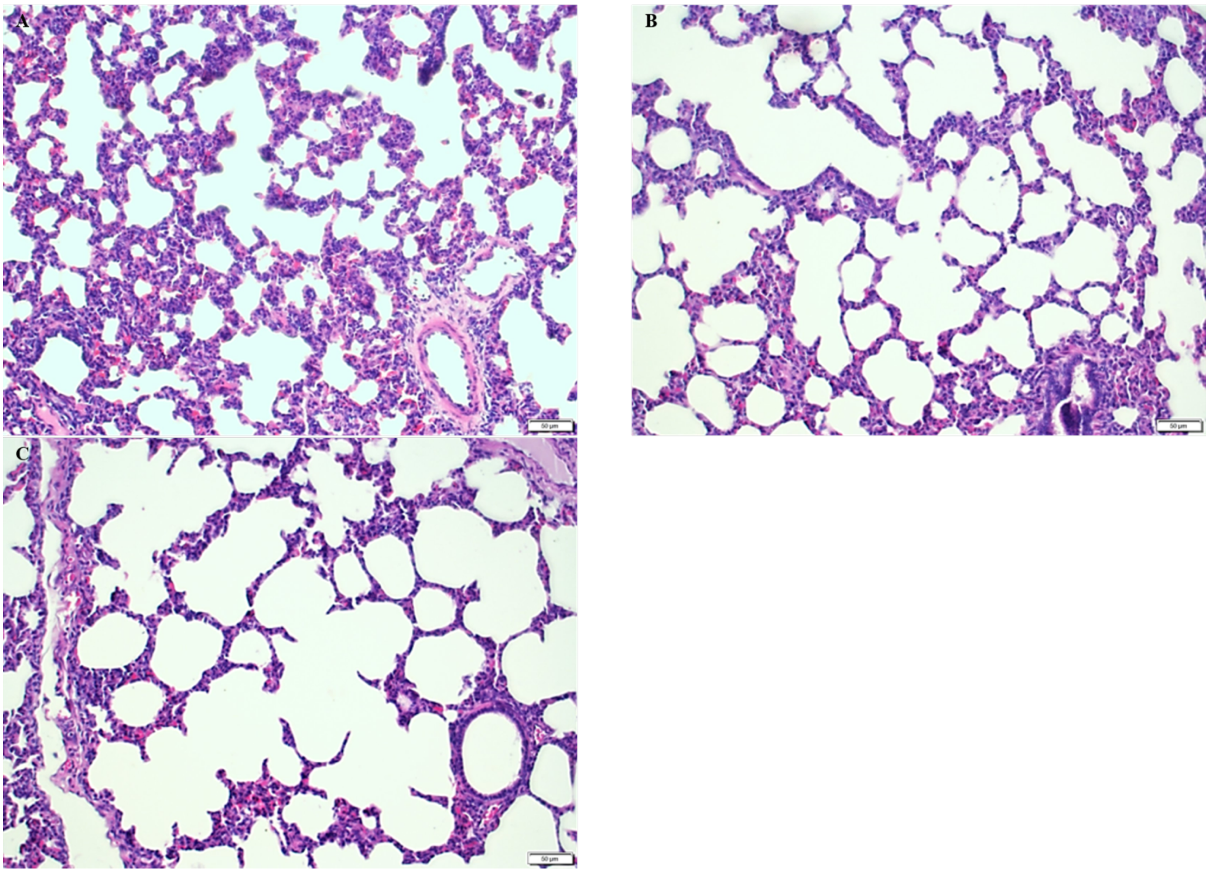

Supplementary Figure S2. Microscopic lung lesion of the unvaccinated challenged (**A**), PRRSV-1 MLV, and PRRSV-2 MLV vaccinated challenged (**B** and **C**) pigs at 7 days post-challenge. H&E staining.

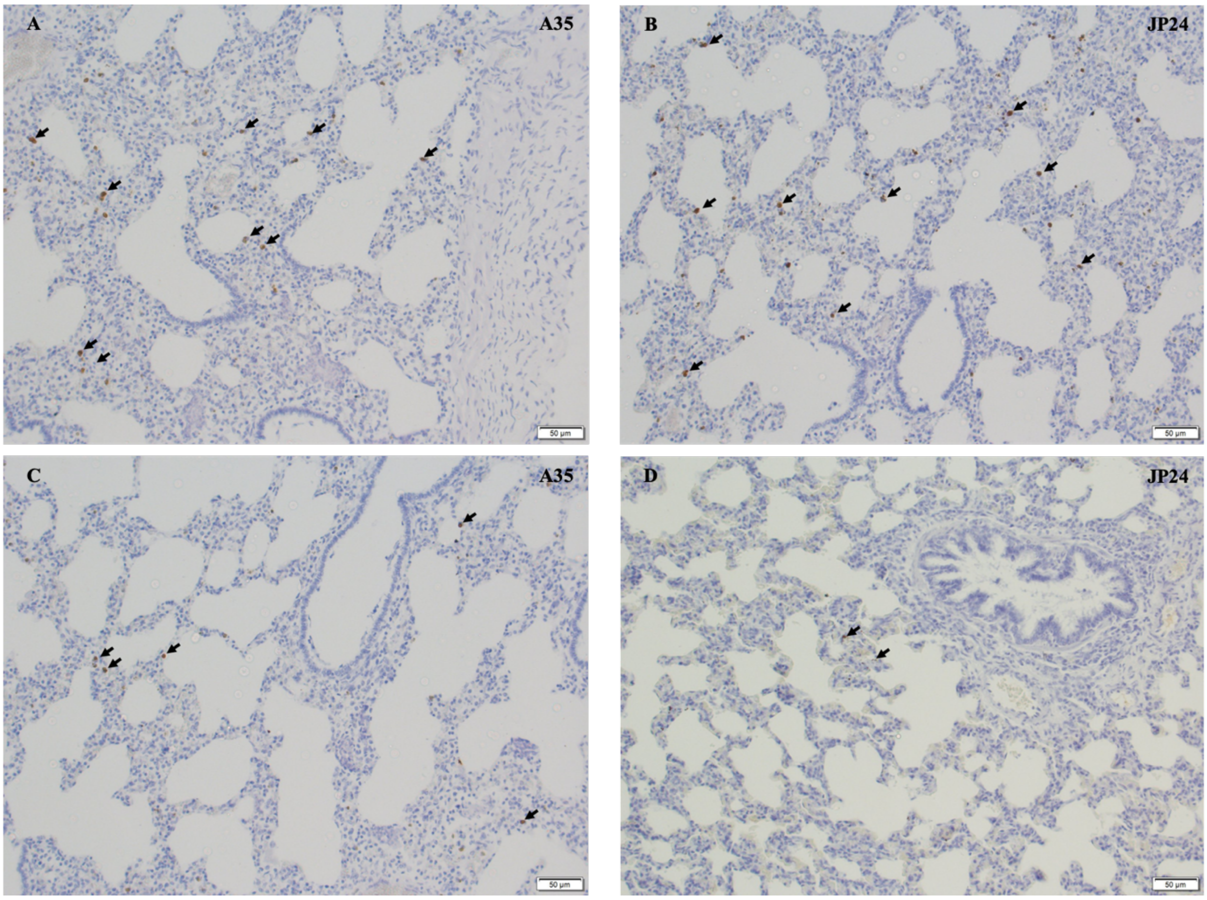

Supplementary Figure S3. Immunohistochemistry of the detection of PRRSV-antigens (arrows) in lung tissues of the unvaccinated challenged (**A** and **B**) and vaccinated challenged (**C** and **D**) pigs at 7 days post-challenge. PRRSV-antigen was detected by monoclonal antibodies against either PRRSV-1 (A35, **A** and **C**) or PRRSV-2 (JP24, **B** and **D**), respectively.
